# Transcatheter valvular therapies in patients with left ventricular assist devices

**DOI:** 10.3389/fcvm.2023.1071805

**Published:** 2023-03-13

**Authors:** Olina Dagher, Marcel Santaló-Corcoy, Nils Perrin, Jean-François Dorval, Neal Duggal, Thomas Modine, Anique Ducharme, Yoan Lamarche, Pierre-Emmanuel Noly, Anita Asgar, Walid Ben Ali

**Affiliations:** ^1^ Department of Surgery, Montreal Heart Institute, Montreal, QC, Canada; ^2^ Department of Cardiac Sciences, Libin Cardiovascular Institute, Calgary, AB, Canada; ^3^ Faculty of Medicine, University of Montreal, Montreal, QC, Canada; ^4^ Department of Cardiology, Montreal Heart Institute, Montreal, QC, Canada; ^5^ Structural Heart Intervention Program, Montreal Heart Institute, Montreal, QC, Canada; ^6^ Cardiology Division, Geneva University Hospitals, Geneva, Switzerland; ^7^ Department of Anesthesiology, Michigan Medicine, Ann Arbor, MI, United States; ^8^ Service Médico-Chirurgical: Valvulopathies-Chirurgie Cardiaque-Cardiologie Interventionelle Structurelle, Hôpital Cardiologique de Haut Lévêque, CHU Bordeaux, Bordeaux, France

**Keywords:** transcatheter heart valve interventions, LVAD (left ventricular assist device), TAVR—transcatheter aortic valve replacement, percutaneous valve intervention, valvular heart disease (VHD), aortic regurgitation (AR), tricuspid regurgitation (TR), mitral regurgitation (MR)

## Abstract

Aortic, mitral and tricuspid valve regurgitation are commonly encountered in patients with continuous-flow left ventricular assist devices (CF-LVADs). These valvular heart conditions either develop prior to CF-LVAD implantation or are induced by the pump itself. They can all have significant detrimental effects on patients' survival and quality of life. With the improved durability of CF-LVADs and the overall rise in their volume of implants, an increasing number of patients will likely require a valvular heart intervention at some point during CF-LVAD therapy. However, these patients are often considered poor reoperative candidates. In this context, percutaneous approaches have emerged as an attractive “off-label” option for this patient population. Recent data show promising results, with high device success rates and rapid symptomatic improvements. However, the occurrence of distinct complications such as device migration, valve thrombosis or hemolysis remain of concern. In this review, we will present the pathophysiology of valvular heart disease in the setting of CF-LVAD support to help us understand the underlying rationale of these potential complications. We will then outline the current recommendations for the management of valvular heart disease in patients with CF-LVAD and discuss their limitations. Lastly, we will summarize the evidence related to transcatheter heart valve interventions in this patient population.

## 1. Introduction

The transition from pulsatile to continuous-flow left ventricular assist devices (CF-LVAD) has allowed significant prolongation of LVAD support because of improved reliability, durability and survival (1). As a result, the therapeutic use of CF-LVADs has expanded from bridge-to-transplant or bridge-to-candidacy strategies to destination therapy for end-stage congestive heart failure. The lack of donor organ availability and the change in organ allocation system are also creating a strong demand for durable LVAD implantation, with prolonged expected waiting time on the transplant list, up to five years even as bridge-to-transplant (2). However, the growing use of LVAD support has been accompanied by a greater recognition of their side effects and complications over time. One of the most noticeable aspects of their adverse events profile is the progression or *de novo* development of valvular heart disease (VHD), particularly aortic insufficiency (3). VHD can negatively impact the quality of life and survival of this patient population (4, 5). The increasing appreciation of the detrimental effects VHD can have on patients with CF-LVAD has led societal guidelines to advocate for a more aggressive management (6).

Many surgical techniques can be used to address these pathologies but they introduce major risks for a patient subgroup who is already particularly vulnerable (7).

Transcatheter heart valves (THV) interventions have revolutionized the management of VHD. The field has evolved rapidly since the first introduction of transcatheter aortic valve replacement (TAVR) for the treatment of aortic stenosis in 2002 (8). Within two decades, indications for THV interventions have extended not only to lower-risk patients, but also to off-label use for patients with no other therapeutic options (9–12). This has stimulated interest in less invasive treatment alternatives for VHD in LVAD patients who are poor surgical candidates. Since the first reported case of TAVR for LVAD-induced aortic insufficiency in 2012, an increasing number of case reports have shown encouraging results (13). Nonetheless, THV interventions in patients with CF-LVAD come with unique decision-making and technical challenges.

After a brief overview of the pathophysiology of VHD in the setting of CF-LVAD support, we will present the current recommendations for their management. This will be followed by a discussion on the available evidence describing THV in patients with CF-LAVD.

## 2. Pathophysiology of valvular heart disease induced by LVAD support

Aortic valve insufficiency, mitral valve insufficiency and tricuspid valve insufficiency are the types of VHD that are most commonly encountered in patients under CF-LVAD support (3). They are either induced by the CF-LVAD itself or precede its implantation.

### 2.1. Aortic valve insufficiency

Unlike mitral regurgitation, aortic regurgitation is rarely present before LVAD implantation (14); when present, it is usually managed at the time of LVAD implant. However, it becomes an increasing concern over time after initiation of LVAD support. More than 25% of patients develop at least moderate aortic regurgitation within the first year of CF-LVAD therapy (15–18). This risk seems to be time-dependent: Cowger et al. showed that between 6 months and 18 months post LVAD, the proportion of patients who had moderate to severe aortic regurgitation went from 11% to 51% (17). The underlying pathophysiology is likely multifactorial, but the most commonly proposed phenomenon is sometimes referred to as the “disuse theory” (18).

The CF-LVAD draws blood from the left ventricle and directs it in parallel into the ascending aorta *via* an outflow cannula, thus bypassing the aortic valve. This effectively increases forward flow and decompresses the left ventricle, but it occurs at the expense of a reduced or absent aortic valve opening. The increased flow in the ascending aorta, coupled with unloading of the left ventricle create a continuous positive transvalvular pressure gradient across the aortic valve, further promoting aortic valve closure. The constant apposition of the coronary cusps stimulates collagen production and proteolytic enzymes activity (19). This eventually leads to leaflet adherence and fusion of the commissures (19). In addition, the high-velocity and turbulent flow in the ascending aorta generates high-shear stress which can cause aortic valve damage and aortic sinus dilation through smooth muscle cell apoptosis (20). The increase in aortic wall stress is directly influenced by the anastomotic angle of the outflow cannula (21). All these factors lead to retraction of the leaflet tips and creation of a fixed central orifice. The pan-cyclic positive transvalvular pressure gradient and high retrograde pressures from the inflow cannula produce a continuous regurgitant jet into the left ventricle. Aortic valve degeneration is further accelerated by thrombus formation on the left ventricular surface owing to the limited antegrade flow and resultant stasis of blood (19). Initiation of CF-LVAD support may also exacerbate pre-existing aortic regurgitation *via* these same mechanisms (22).

The “disuse theory” has been supported by observational studies. It was found that aortic regurgitation occurs 6 times more frequently in patients in whom the aortic valve remains closed compared to patients with frequent aortic valve opening (16).

The physiologic and clinical consequences of aortic regurgitation are significant. The regurgitant blood is diverted from the left ventricle into the pump. The pump then propels this blood forward to the ascending aorta, but it returns back into the left ventricle down the continuous pressure gradient owing to the aortic insufficiency. This creates a closed loop circulation, rendering the CF-LVAD output ineffective (23). Recirculating blood in the left ventricle also increases left ventricular volumes and pressures, which leads to recurrence of heart failure symptoms (24). Early identification and evaluation of the severity of aortic insufficiency under CF-LVAD support may be challenging since traditional echocardiographic criteria, which are based on diastolic volume overload, markedly underestimate the severity of this regurgitation (21). Although small amounts of aortic regurgitation can be tolerated, more severe regurgitation requires intervention as it can lead to end-organ malperfusion (17).

### 2.2. Mitral valve insufficiency

Most cases of mitral insufficiency in LVAD patients are functional in nature and can be attributed to ventricular remodeling and left ventricular dilatation present prior to LVAD implantation (3). Close to 60% of CF-LVAD recipients have at least moderate mitral regurgitation at the time of CF-LVAD implantation (25). However, unlike aortic regurgitation, the severity of mitral regurgitation can be decreased with the use of CF-LVAD alone: left ventricular decompression reduces left ventricular dimensions and increases mitral leaflet coaptation (26). In a retrospective study of 100 consecutive CF-VADs, Morgan et al. showed that CF-LVAD significantly decreased the proportion of patients having moderate or severe mitral regurgitation from 76% at 1 month to 8% at 6 months (26). Unfortunately, this beneficial effect of CF-LVAD support does not occur in all patients. In a single-center retrospective analysis, persistent moderate or severe mitral regurgitation was still observed in 26% of patients after 6 months of CF-LVAD therapy (27). The absence of improvement in mitral regurgitation could be explained by suboptimal left ventricular decompression. Several factors can prevent effective ventricular unloading: significant aortic regurgitation, intractable fluid retention, inadequate pump speed, inadequate pump position, pump thrombosis or frequent suction events. These factors cannot always be corrected without an invasive intervention. For example, malalignment of the apical inflow cannula could result in an obstruction of its orifice owing to an inward bowing of the interventricular septum or ventricular free wall. This situation usually warrants surgical intervention (28). Several studies have documented the effects of residual mitral regurgitation after CF-LVAD implantation. It was found to be associated with persistent pulmonary hypertension, worse right ventricular hypertension, higher risk of renal failure, repeat hospitalizations and increased mortality (29–31).

### 2.3. Tricuspid valve insufficiency

Tricuspid insufficiency in patients under LVAD support is typically functional (32). One-third to two-thirds of patients with advanced heart failure will have associated tricuspid insufficiency (32). Functional tricuspid regurgitation (TR) often appears in conjunction with left-sided valve disease and left ventricular dysfunction despite the presence of a structurally normal tricuspid valve. It is caused by dilatation of the right ventricle with secondary annular enlargement, apical leaflet displacement and resultant tethering and incomplete coaptation. This condition triggers a vicious cycle where tricuspid regurgitation further contributes to right ventricular failure through right-sided volume overload and decreased ejection. The evolution of tricuspid regurgitation under CF-LVAD support can take many forms. On one hand, by allowing mechanical unloading of the left ventricle, CF-LVAD support decreases left ventricle end-diastolic pressure and pulmonary venous pressure which, in turn, decreases right ventricular afterload (3). Since the right ventricle is highly afterload-sensitive, a decrease in pulmonary pressures improves right ventricular contractility and decreases right ventricular end-diastolic dimensions, as well as tricuspid annular diameter. On the other hand, by reducing left ventricular volumes, the CF-LVAD can also acutely exacerbate tricuspid regurgitation (3). First, it may cause a leftward shift of the interventricular septum, resulting in a restriction of the tricuspid leaflets (33). Second, as systemic flows improve with CF-LVAD support, venous return and right ventricular preload also increase, which can worsen an already marginal right ventricular function and tricuspid regurgitation (34). Given the opposing effects of CF-LVAD on tricuspid regurgitation, progression of this VHD is difficult to predict in patients with CF-LVAD. Furthermore, patients with chronic heart failure often develop pulmonary hypertension from pulmonary arterial vasoconstriction and remodelling resulting from the chronically increased left-heart filling pressure (35). Although pulmonary hypertension usually improves with CF-LVAD support, it may not be normalized in all patients (36). Residual pulmonary hypertension in patients under CF-LVAD support contributes to right-sided heart failure, and thus secondary tricuspid regurgitation (34).

In a retrospective study of 127 patients with over 1 year of LVAD support, the incidence of moderate to severe residual tricuspid regurgitation was found to be 24% (37). The regurgitant fraction impairs transpulmonary flow, thereby reducing CF-LVAD filling and systemic output (38). In addition, the increased diastolic pressure in the right ventricle can cause septal shift and compression of the left ventricle, which may also reduce LVAD filling (38). Systolic reversal of flow in the vena cava may be responsible for end-organ venous congestion. This could negatively affect liver and renal functions, to the point where cardiac transplantation alone becomes contraindicated. Uncorrected moderate or severe tricuspid regurgitation is associated with increased duration of inotropic support and hospitalization, increased rates of right ventricular assist devices, as well as decreased survival in patients with CF-LVAD (32, 39).

## 3. Indications for intervention

The most recent recommendations for the management of VHD in the context of anticipated long-term LVAD support were published in 2013 by the International Society for Heart and Lung Transplantation (6). They are only based on expert opinion or small retrospective studies and concern mainly management of VHD at the time of LVAD implantation.

### 3.1. Aortic valve

Given that aortic regurgitation almost invariably progresses under CF-LVAD support and given its significant hemodynamic and clinical consequences, addressing moderate to severe aortic regurgitation at the time of LVAD implantation is a class I indication (level of evidence (LOE) C) (6). In the presence of any concomitant aortic stenosis, bioprosthetic aortic valve replacement should be favored over other surgical interventions (Class I, LOE C) (6). Pre-existing aortic stenosis is less of a concern since pump output does not depend on aortic valve opening. Accordingly, aortic valve replacement for severe aortic stenosis is a class IIb recommendation (LOE C) (6).

### 3.2. Mitral valve

Since there is an expected improvement in mitral valve regurgitation under CF-LVAD support, recommendations for the surgical management of mitral regurgitation remain conservative. As per these international guidelines, routine surgical intervention for severe mitral insufficiency is not recommended, unless there is expectation of cardiac recovery (class III, LOE C) (6). Significant mitral stenosis impairs left ventricular filling, thus CF-LVAD filling. Guidelines therefore recommend a mitral valve replacement at the time of CF-LVAD implant for at least moderate mitral stenosis (6). Other authors have suggested considering concomitant mitral valve intervention in the following select scenarios: (i) patients with severe mitral regurgitation and pulmonary hypertension who are bridge-to-transplant or bridge-to-candidacy, (ii) severe mitral regurgitation with posterior displacement of the coaptation point, and (iii) destination therapy patients with borderline right ventricular function (34).

### 3.3. Tricuspid valve

Guidelines advocate a liberal approach to concomitant tricuspid valve repair in the presence of moderate to severe tricuspid regurgitation with long-term LVAD support (class IIa, LOE C) (6). However, this approach is still controversial and largely debated. Recent studies, including one randomized clinical trial—the TVVAD trial (NCT03775759) –, found that concurrent tricuspid valve repair at the time of LVAD implantation does not appear to lower the incidence of right heart failure (40–42).

Only one study compared concomitant tricuspid valve repair and replacement during LVAD implant (40). After a mean time of 12.3 ± 9.7 months, they found that late mortality and the magnitude of reduction in regurgitation severity was similar between groups.

## 4. THV interventions

While current guidelines address the presence of VHD at the time of LVAD implantation, there is no consensus yet on the management of VHD once LVAD support has been initiated. While for the majority of cases, tricuspid or mitral regurgitation may be dealt with at the time of LVAD implantation, aortic regurgitation develops as a consequence of LVAD support. In addition, previously implanted prosthetic valves might eventually fail over time. This is especially true for bioprosthetic valves in the aortic position since they are prone to the disuse phenomenon (43). With the overall increase in CF-LVAD support duration, more and more patients may reach a point where valve intervention is deemed necessary. Conventional surgical procedures are certainly feasible, but they hold a high risk of complications (34). Patients with CF-LVAD often have a high burden of comorbidities, a history of prior sternotomy and poor right ventricular function. Therefore, there is an understandable reticence to prolong CF-LVAD surgery or to expose these patients to another invasive cardiac surgery after CF-LVAD implant. In this context, transcatheter heart valve interventions appear as an attractive alternative. However, literature detailing these approaches is largely confined to case reports and small case series (Table 1).

**Table 1:** Percutaneous devices used so far and potential complications of transcatheter heart valve interventions under left ventricular assist device support.

Valve	Percutaneous devices used so far	Number of reported cases	References	Reported complications	Ways to prevent or to manage
Aortic	CoreValve, Evolut R, Evolut Pro	41	(44–63)	Valve migration	Valve oversizing, valve-in-valve, valve-in-ring, self-fixating prosthesis
	Sapien, Sapien XT, Sapien 3	17	(13, 46, 47, 55, 64–72)	Paravalvular leak	Valve oversizing, balloon overinflation (for balloon-expandable models), valve-in-valve
	ACURATE Neo	1	(73)		
	JenaValve	1	(74)	Valve deterioration by disuse	Ramp study to allow at least partial aortic valve opening
	Melody	1	(43)	Valve thrombosis	Ensure optimal anticoagulation, prompt recognition and diagnosis
	Unknown (TAVR)	87	(75)		
	Amplatzer Occluder	34	(46, 76–89)	Hemolysis (with septal occluders)	Avoid peridevice regurgitant flow
Mitral	MitraClip	33	(90–92)	Increased transvalvular pressure gradients/iatrogenic mitral stenosis	Appropriate patient selection to identify best suited valve morphology, Avoid excessive adduction of the anterior and posterior leaflets
	Valve-in-Valve (Sapien XT)	1	(52)	Inter-atrial shunt	Avoid placing >2 MitraClips, Percutaneous ASD closure
Tricuspid	MitraClip XTR		(93)	Residual regurgitant jet	Additional clips deployment
				Leaflet tear	Caution with MitraClip G4 systems
				Conduction abnormalities	Avoid excessive radial strain with valve deployment
		1		Single-leaflet device attachment	Additional clips deployment
				Valve thrombosis	Ensure optimal anticoagulation, prompt recognition and diagnosis
				Stent migration (TriCinch or CAVI)	Avoid if bridge-to-transplant or if vena cava are too dilated

ASD: atrial septal defect, CAVI: caval vale implantation, TAVR: transcatheter aortic valve replacement.

### 4.1. Aortic valve

Transcatheter options include TAVR and aortic valve closure with a percutaneous septal occluder.

#### 4.1.1. TAVR

TAVR has been widely adopted for aortic stenosis. It has also been established as a therapeutic option for aortic insufficiency from degenerative bioprosthesis (94). However, several important technical challenges have limited the suitability of TAVR for native aortic insufficiency. With or without CF-LVAD, native aortic insufficiency typically exists in non-calcified valves. Most TAVR systems rely on the radial tension applied by the prosthesis on the aortic complex, as well as on the interaction of the stent frame with aortic calcifications for proper anchoring. In the setting of aortic insufficiency induced by LVAD, the lack of calcifications may compromise prosthesis stability (95). Furthermore, the presence of aortic root dilation is not uncommon in patients under CF-LVAD (96). These changes can occur as early as within the first 6 months after initiation of CF-LVAD support and are thought to be caused by an increase in aortic wall sheer stress (64). The absence of an anchoring support leads to an increased risk of device misplacement or migration and paravalvular regurgitation from an incomplete seal (95). These can be corrected by using a valve-in-valve strategy in which a second valve is delivered to hold the first valve in place and prevent its migration (44, 64). If not properly anchored, the prosthesis could migrate into the left ventricular outflow tract or the left ventricle, where it could obstruct the inflow cannula of the CF-LVAD. These catastrophic scenarios occur suddenly and require emergent sternotomy and salvage surgery (44, 45). The risk of migration during TAVR deployment is further increased by the hydrodynamic forces exerted by the LVAD: on one hand, the inflow cannula creates a continuous suction effect towards the apex of the left ventricle, and on the other hand, the flow from the outflow cannula causes an opposing force against the valve during its deployment. To minimize this risk, LVAD flows should be temporarily decreased or turned off during valve deployment (44).

In patients with pure aortic regurgitation who are not under CF-LVAD support, systematic reviews from observational studies showed promising results with TAVR, especially with newer generation systems, despite the need to perform a valve-in-valve procedure in 7 to 30% of cases (95, 97, 98). In a meta-analysis of 12 studies, representing a population of 638 patients, Haddad et al. compared the short-term outcomes of non-LVAD patients with pure native aortic regurgitation who underwent TAVR between 2007 and 2016 (98). Mean logistic EuroScore II was 11.7 ± 12.9% in first generation valves and 9.3 ± 6.4% in second generation valves (98). The mean STS score in first generation valves was 13.1 ± 2.0% compared to 9.1 ± 3.6% in second generation valves (98). The rate of device success was 92% (95% CI from 83% to 99%) in second generation valves compared to 68% (59%–77%) for first generation valves (98). The occurrence of residual moderate or severe aortic regurgitation went from 16% (6%–29%) with first generation valves to 1% (0%–5%) with second generation valves (98). Conversion to surgical aortic valve replacement was also lower in second generation valves, 1% (95% CI from 0% to 4%), compared to first generation valves, 2% (95% CI from 0% to 6%) (98).

Newer generation transcatheter aortic valves offer many benefits, including repositionability, self-positioning geometry, and specific fixation mechanisms, that have the potential to improve the performance of TAVR in patients with native aortic regurgitation (95). The JenaValve (JenaValve Technology, Inc., Munich, Germany) and the ACURATE TA (Symetis, Ecublens, Switzerland) are currently the only devices with *Conformité Européenne* mark for the treatment of aortic regurgitation. They also received approval from the United States Food and Drug Administration for the conduct of clinical trials. The JenaValve is a self-expanding porcine valve on a nitinol frame made of three integrated feelers (also called locators). The locators allow to align the device with native aortic valve anatomy and clip onto the native leaflets which forms a natural seal and fixation independent of valve calcification (99). The first case series of TAVR in aortic regurgitation using the JenaValve showed a procedural success of 97%–100% (100–102). Ranard et al. recently reported the first use of the JenaValve to address severe aortic regurgitation in a CF-LVAD patient (74). The procedure was uncomplicated and there was no transvalvular or paravalvular leak after deployment of one prosthesis. The ACURATE TA system (Symetis, Ecublens, Switzerland) features a self-fixing mechanism made of two crowns, suitable for larger annuli, but requires transapical access (99). Other promising systems include Direct Flow Valve System (Direct Flow Medical, Santa Rosa, California), J-Valve (Jie Cheng Medical Technologies, Suzhou, China), Engager valve (Medtronic Inc., Minneapolis, MN, United States) and the Lotus valve (Boston Scientific, Natick, Massachusetts) (99).

While awaiting the approval of these devices, the CoreValve (Medtronic Inc., Minneapolis, MN, United States) and Sapien (Edwards Lifesciences Inc., Irvine, California) systems have shown good results (46). However, given the paucity of data on TAVR in patients supported with LVADs, there is a lack of consensus on which of the two systems is better suited for this off-label use. The CoreValve has the advantage of aortic fixation, while the SAPIEN, with its balloon-expandable deployment, applies enhanced radial force on the ring and is associated with lower paravalvular leakage. Some authors deliberately oversize the valve to further increase radial pressure and improve anchoring. In the first reported case of TAVR in a patient with LVAD, D'Ancona et al. used a 29 mm SAPIEN valve within a 21 mm annulus that would normally require a 23 mm valve (13). Pal et al. successfully deployed a 31 mm CoreValve -oversized by 17%- followed by a 29 mm valve-in-valve SAPIEN-3 in two patients with CF-LVAD (47). The CoreValve fixation within the aorta served as a scaffold to anchor the SAPIEN-3 in the absence of annular calcifications, while the SAPIEN-3 eliminated paravalvular leakage once overinflated. However, due to the costs associated with the use of two prostheses, this technique cannot be universally used. In our local practice, we favor the CoreValve over the Sapien valve. The ability to recapture and reposition the CoreValve at up to 80% deployment is very advantageous in this clinical context. After positioning the valve, it is deployed right before the point of no recapture and the pump speed is increased slowly. The operator then ensures that the valve remains stable before completing deployment, reducing the risk of valve embolization.

In patients under LVAD support with a history of aortic valve replacement, the prosthesis itself can be used for anchoring. Yap et al. used a 26 mm SAPIEN-3 in a structurally deteriorated 29 mm Toronto Freestyle (65). The fibrotic response at the sewing ring provided sufficient resistance to allow proper anchoring and the TAVR was placed in a subannular position. Chung et al. proposed a novel solution by placing an internal aortic annuloplasty ring in a patient with mild aortic regurgitation at the time of LVAD implantation, which could serve as an anchor for subsequent TAVR, in the event of progressive aortic regurgitation (48) but this strategy needs to be further studied.

Regarding access planning for TAVR in patients with aortic regurgitation, the transapical approach is most commonly used (42%–55% of cases), closely followed by transfemoral approach (39%–41% of cases) (95, 97). Any TAVR procedure relies on the measurements of aortic annulus, aortic root, and iliofemoral anatomy for access planning and valve selection. Patients under LVAD with end-stage ischemic heart disease may have significant peripheral vascular disease, precluding transfemoral access. Transapical access has been reported, but care should be taken not to compromise the LVAD inflow cannula (13). A preoperative chest computed tomography helps identify a safe route for access through the apex (13). TAVR deployment directly *via* the LVAD inflow cannula has also been done (66). This approach was performed in the context of concomitant LVAD pump exchange, which already required initiation of cardiopulmonary bypass and LVAD pump removal (66). Because of the short length of the LVAD inflow graft, a Dacron graft was anastomosed end-to-end to the LVAD inflow graft in order to provide additional working length for the placement of the large bore sheath of the TAVR (66).

Transcatheter valves are also vulnerable to LVAD-induced structural deterioration. Derryberry et al. described complete fusion of an Evolut R leaflets within 5 months of LVAD support (49). The patient was a bridge-to-transplant and was therefore not affected by this premature deterioration. Parry et al. reported complete fusion of a CoreValve 33 days after implantation in a 64-year-old patient with a “bridge-to-recovery” scenario (50). This complication was discovered in the operating room while attempting LVAD explant. Less than a week later, the patient died from an extensive stroke. Autopsy revealed the presence of organizing thrombus covering the ventricular surface of the CoreValve with overlying recent thrombus, despite appropriate anticoagulation therapy (50). This unfortunate case demonstrates that the unique physiology of an LVAD might lead to worrying complication and early TAVR deterioration. Efforts to maintain regular valve opening by running the LVAD at lower speed than usual might prevent bioprosthetic aortic valve thrombosis, although cusps may be difficult to visualize due to the metal frame of the TAVR. If that is the case, a high pulsatility index may indicate probable opening of the aortic valve. Transesophageal echocardiography or multiphase computed tomography can be performed in case of high suspicion for thrombosis and inconclusive transthoracic echocardiogram, such as in the event of increasing valvular gradients or visual thickening of the valve (51). Noteworthy, the recent recommendations for the management of antithrombotic therapy in patients undergoing TAVR do not mention LVAD patients (103). After diagnosing TAVR thrombosis in a LVAD patient, Rao et al. started unfractionated heparin and increased LVAD speed to minimize risk of aortic valve opening, and therefore attempt to mitigate the risk of embolization (51). However, intravenous anticoagulation was complicated by gastrointestinal bleeding requiring multiple transfusion and the high LVAD speed could not be maintained due to persistent suction events (51). Luckily, the patient remained stable while awaiting transplantation (51). This case illustrates the therapeutic dilemma in patients with LVAD who develop TAVR thrombosis.

#### 4.1.2. Percutaneous transcatheter aortic valve closure

Percutaneous transcatheter aortic valve closure with a septal occluder such as the Amplatzer Multi-Fenestrated Septal Occluder device (St Jude Medical, Saint Paul, MN), is essentially the same as a surgical left ventricular outflow closure in which the aortic valve is oversewn. However, similarly to TAVR, this procedure has only been reported in a few small case series, with a lack of long-term data (76–86). Potential complications include device migration, thrombus formation, hemolysis from peri-device regurgitant flow, erosion in aorto-mitral curtain and coronary ostia obstruction. In order to reduce the risks of residual shunting, hemolysis, and device embolization, some authors have been using an oversizing strategy (87). Acceptable results have also been reported with smaller device/annulus ratios (88).

Despite the lack of sufficient data to perform a comparative analysis between TAVR and transcatheter aortic valve closure, the latter technique was found to have a higher mortality rate compared to TAVR (46, 88). In addition, closing the aortic valve results in complete LVAD dependency, which could be fatal in case of sudden power loss, pump thrombosis or other mechanical failure. For all these reasons, the use of percutaneous transcatheter aortic valve closure in LVAD patients has been almost exclusively abandoned in favor of TAVR.

### 4.2. Mitral valve

In 2020, the American College of Cardiology/American Heart Association (ACC/AHA) Valvular Heart Disease Guidelines incorporated transcatheter edge-to-edge repair using the MitraClip (Abbott Vascular, Santa Clara, California) as a Class IIa recommendation for intervention for secondary mitral regurgitation in patients with persistent severe symptoms despite treatment with guideline-directed medical therapy (104). Therefore, it is expected that an increasing number of CF-LVAD candidates will have a MitraClip in place at the time of CF-LVAD implantation. A number of case reports and small case series have documented the feasibility and safety of LVAD implantation in patients with prior MitraClip (105–108). Transcatheter mitral valve repair did not impact hemodynamic nor mortality in patients with LVAD (105–108). On the other hand, in patients with end-stage heart failure and secondary mitral regurgitation, it remains unclear whether MitraClip has any value as a bridge to transplant or bridge to LVAD and whether it would be more beneficial to perform a mitral valvuloplasty at the time of LVAD implantation (109–111).

A few cases of MitraClip procedures performed following CF-LVAD implantation have been reported (90–92). The largest series was comprised of 30 patients (92). In this registry study, Tanveer et al. compared the short-term outcomes of patients with LVAD who underwent MitraClip (*n* = 30) vs. surgical mitral repair (*n* = 199) between 2016 and 2018 in the United States (92). Patients who underwent MitraClip implantation were older on average (61.2 vs. 56.4 years, *p* = 0.223) and had a higher prevalence of renal failure, peripheral vascular disease, atrial fibrillation, smoking history and previous permanent pacemaker/implantable cardioverter defibrillator. In-hospital mortality was higher in the MitraClip group (Cell count < 11 out of 30 patients for MitraClip vs. 6.9% for surgical repair). Nonfatal complications including acute kidney injury, bleeding requiring transfusion and vascular complications were lower in the MitraClip group. Patients who underwent MitraClip intervention also had, on average, a shorter hospital stay and lower hospital costs. Given the small sample size and retrospective nature of the study, these findings remain hypothesis-generating, but they certainly show a promising

potential for MitraClip in patient with LVAD.

Some authors questioned whether the presence of >2 MitraClips -an uncommon event- could negatively affect LVAD therapy by: (i) affecting right ventricular function due to the presence of an inter-atrial shunt through the residual atrial septal defect and (ii) decreasing left ventricular filling and LVAD flow as a result of a reduced valve area combined with the increased flow rates after LVAD initiation (112, 113). Raghunathan et al. reported a case of bidirectional -predominantly right-to-left- shunting immediately after the delivery of two MitraClip XTR in an LVAD patient with right ventricular dysfunction (91). Percutaneous closure of the transseptal puncture with an Amplatzer occluder device led to symptomatic improvement of the patient's heart failure symptoms (91). Of note, less than 30% of patients have a residual iatrogenic atrial septal defect 1 year from MitraClip treatment (114). This proportion is however unknown in the presence of an LVAD and abnormal right-sided pressures. Meduri et al. reported a successful mitral valve replacement in a patient with LVAD and severely stenotic bioprosthetic mitral valve. A 29 mm Sapien XT was deployed in a stenotic 27 mm Carpienter-Edwards Perimount mitral prosthesis (Edward Lifesciences, Irving, CA) *via* transseptal access (52). The patient also developed symptoms from a significant right-to-left shunt secondary to iatrogenic atrial septal defect and required percutaneous closure (52). While these adverse events may seem anecdotal, they should be investigated further.

From a procedural standpoint, care should be taken not to damage the LVAD inflow cannula when crossing the mitral valve with the mitral valve devices. In addition, the LVAD can create a prominent “waterfall” color Doppler artifact, interfering with the evaluation of transmitral flows pre- and post-procedure (115).

### 4.3. Tricuspid valve

Currently used tricuspid valve catheter devices can be divided into four categories, according to their mode of action: annuloplasty devices, edge-to-edge repair devices, valve replacement devices and caval valve implantation (116). The type of device should be tailored to the underlying mechanism of tricuspid valve disease and should take into account the presence of pacemaker leads. The indication for LVAD implantation should also be taken into account when choosing the optimal transcatheter tricuspid valve procedure. For example, an edge-to-edge repair might be favored over a replacement approach as an acceptable shorter-term solution in bridge-to-transplant or bridge-to-recovery therapies while avoiding the extra costs of transcatheter bioprosthesis. In a bridge-to-transplant strategy, the TriCinch system (4Tech Cardio, Galway, Ireland) or caval valve implantation would not represent an optimal solution due to the risk of stent migration in the inferior vena cava during the heart transplantation. Transcatheter tricuspid technologies are still under preclinical or initial clinical evaluation but early safety and feasibility trials conducted to date have shown promising results (117). In addition, tricuspid valve regurgitation is typically addressed at the time of LVAD implantation. These factors explain the lack of data on percutaneous tricuspid valve interventions in patients with CF-LVAD. Furthermore, multivariate analysis of the Transcatheter Tricuspid Valve Therapies (TriValve) registry data revealed the existence of multiple factors associated with lower procedural success, independent of the device used: increased coaptation depth, larger annular diameter and increased pulmonary artery pressure (118). Although patients from this registry were not under LVAD support, these results suggest that transcatheter tricuspid interventions should be performed earlier, preceding the development of severe right ventricular remodeling, in order to increase the chance of procedural success. Therefore, in patients with CF-LVAD who frequently have some degree of right ventricular dysfunction, the optimal timing to address the tricuspid regurgitation might very well be at the time of LVAD implantation.

To our knowledge, only one case report to this date has described a transcatheter tricuspid valve intervention on a patient under LVAD support (93). The patient was a 59-year-old female who previously underwent a HeartMate III implantation and tricuspid annuloplasty with a 32 mm rigid ring as a bridge to transplantation. After two months, she developed recurrent severe tricuspid regurgitation with right ventricular decompensation needing continuous inotropic support. The cause of the tricuspid regurgitation was identified as being a partial detachment of the prosthetic ring. She was successfully treated with a transcatheter edge-to-edge repair using the MitraClip XTR system. A first device was used to clip the antero-septal commissure, and, because of a residual regurgitant jet, a second clip was placed between the septal and the posterior leaflet. This is reminiscent of the “triple orifice technique” or the “clover technique” originally described in conventional tricuspid repair surgery (119, 120). Using these two MitraClips, the tricuspid regurgitation was reduced by 50%, leading to a postoperative effective regurgitant orifice area of 0.7 cm^2^ (93). The patient's demands for inotropic support stabilized and she was successfully transplanted 30 days after the clipping procedure (93).

The off-label use of the MitraClip has been the first-choice approach for high-risk patients with secondary tricuspid regurgitation, likely because of wide availability and operator familiarity with the device (11). However, steering the MitraClip through the right atrium remains challenging given the anatomic obstacles inherent to right-sided interventions and its use in tricuspid procedures will certainly become obsolete with the commercial availability of the dedicated TriClip system (Abbott Vascular, Santa Clara, California) (121). The TriClip has a similar configuration as the MitraClip, including a clip delivery system and a steerable guide. It's safety and efficacy were recently shown in the TRILUMINATE trial (121). Moreover, the next-generation TriClip G4 system (NT, XT, NTW, and XTW) has wider clip arms and allows independent leaflet capture, which should facilitate leaflet grasping even in the presence of broader coaptation gap. The TriClip and TriClip G4 are currently being tested in the TRILUMINATE Pivotal Trial (NCT03904147). The rate of single-leaflet device attachment will certainly deserve attention as large coaptation gaps are commonly found in patients with functional TR and advanced ventricular remodeling, often requiring multiple grasping attempts and clips.

Regarding transcatheter tricuspid valve replacement, many devices are actively being studied in early feasibility trials (116). Factors that could hinder prosthetic valve positioning in patients with CF-LVAD include the lack of annular calcifications, a large annular size and the presence of pre-existing cardiac implantable electronic devices (11). Improper anchoring may lead to device malfunction, paravalvular leak, valve embolism, or valve thrombosis. On the other hand, too much radial strain could compromise the atrioventricular node or bundle of His and lead to conduction abnormalities. Indeed, conduction abnormalities seem to occur more frequently with transcatheter tricuspid valve replacement compared to surgical or transcatheter repair (122). In addition, since right-sided valves are exposed to low pressures and low velocity flows, the risk of valvular thrombus formation is believed to be higher than that of left-sided valves (123).

The unique procedural challenges related to the characteristics of the tricuspid valve (leaflet fragility, large non calcific annulus, angulation in relation to the vena cava, presence of chief surrounding structures) make multimodality imaging key not only for preprocedural planning, but also for intraprocedural monitoring. In patients with LVAD, imaging guidance might be limited by the presence of shadowing or artefacts. Transesophageal echocardiography imaging might also be limited by the anterior location of the tricuspid valve, making the use of intracardiac echocardiography an appealing alternative (37).

## 5. How do THV interventions compare to surgery in CF-LVAD patients?

Patients under LVAD support have a high-risk profile and may be deemed unfit to sustain conventional redo open-heart surgery, which involves general anesthesia, endotracheal intubation, cardiopulmonary bypass and aortic cross clamping. In this context, percutaneous approaches are, understandably, an attractive “off-label” option. However, the techniques are still in their infancy and there is still a lot of uncertainty regarding long-term outcomes.

Few observational studies compared the short-term and midterm outcomes of secondary surgical aortic valve replacement (SAVR) vs. TAVR in patients with CF-LVAD. Zaidi et al. conducted a retrospective analysis of all relevant patient information extracted from the Nationwide Readmission Database in the United States between 2016 and 2018 (75). A total of 148 patients were included, 87 in the TAVR group and 61 in the SAVR group. The inpatient mortality in the SAVR group was numerically higher compared to the TAVR group, but did not reach statistical significance (<16% vs. <8%, adjusted odds ratio (aOR) 2.45, confidence interval (CI) 0.41–14.7, *p* = 0.32). Mean length of hospital stay was significantly higher in the SAVR group (40 vs. 13.8 days, aOR 19.9, CI 9.65–30.1, *p* < 0.001). Thirty-day all-cause readmission rate, cardiogenic shock, bleeding and vascular complications were also higher in the SAVR group compared to the TAVR group. Rali et al. reviewed patients from the National Inpatient Sample database from 2015 to 2018 (124). During the study period, a total of 105 TAVR implantations and 50 SAVR procedures were performed in LVAD patients. Patients undergoing TAVR were older but had a lower comorbidity index compared to the SAVR group. They were also more likely to undergo the procedure electively. The difference in baseline characteristics is counter-intuitive since one would expect TAVR to be favored in sicker patients. This might reveal a certain hesitation from surgeons and interventionalists to use an emerging off-label technique in higher risk situations. The composite outcome of in-hospital mortality, stroke, transient ischaemic attack, myocardial infarction, pacemaker implantation, need for open aortic valve surgery, vascular complications and cardiac tamponade was higher among patients undergoing SAVR (30%) compared with those undergoing TAVR (14%), including after multivariable adjusted analyses (aOR 0.24; 95% CI [0.06–0.97]; *p* = 0.045). The prevalence of postprocedural moderate-to-severe paravalvular regurgitation (TAVR: 14%; SAVR: 0%), acute kidney injury (TAVR: 33%; SAVR: 60%) and bleeding requiring transfusions (TAVR: 0%; SAVR: 20%) did not significantly differ between the two groups after adjustments in the multivariable model.

The long-term outcomes of TAVR in LVAD patients are very limited. A small single-center study reported a one-year survival post TAVR of 73% (125). All survivors experienced an improvement in their left ventricle end-diastolic diameter (mean reduction of 6.8 ± 4.4 mm), NYHA functional class and the Kansas City Cardiomyopathy Questionnaire score at 1 year. For almost two-thirds of these patients, the improvement in their Kansas City Cardiomyopathy Questionnaire score was >20 points. The long-term outcomes of secondary SAVR in LVAD patients have also been poorly investigated. In a single-center study that included 6 LVAD patients undergoing secondary SAVR between 2009 and 2020, survival was 67% after a median follow-up of 29 (6–64) months (126). Causes of death were pneumogenic sepsis 1 month after surgery and immune reaction following heart transplant. It is worth nothing that survival rates and long-term outcomes are difficult to compare in LVAD patients since they are strongly influenced by the heart failure status and management, the etiology, the support strategy (bridge to transplant, destination strategy, etc.) and occurrence of heart transplant. Gathering long-term outcome data on patients with LVAD for destination therapy is important not only because they constitute a rising proportion of LVAD recipients, but also because it would inform as to whether TAVR and/or secondary SAVR improve the prognosis of the underlying cardiomyopathy. Until then, the evidence so far, albeit scarce, suggests that both SAVR and TAVR are viable treatment options for aortic regurgitation in patients with CF-LVAD. With TAVR, the risk of device migration and significant postimplant paravalvular leak should be kept in mind, owing to the intrinsic anatomical and technical challenges presented previously. With SAVR, postoperative morbidity such as stroke, significant bleeding or right heart failure remain a concern. There are currently no guidelines that recommend one approach over the other. Some authors have suggested choosing TAVR over secondary SAVR in destination therapy patients or bridge-to-transplant patients who are faced with long waiting times (125, 126). TAVR might also be used in certain emergent/rescue interventions: Wilson et al. reported a successful emergent TAVR in a patient with a fused aortic valve who suffered a cardiac arrest as a result of sudden LVAD pump failure (53). On the contrary, SAVR might be a better option in patients for whom heart transplantation is closely available, especially in the presence of aortic root dilation and absence of valve calcification,

Finally, no study to date has compared mitral or tricuspid valve replacement with THV interventions and long-term outcomes are largely unknown. However, these devices are not exposed to the same hemodynamic environment as the aortic valve, and the risks of sudden, catastrophic intraprocedural complications are less of a concern compared to TAVR.

## 6. What's next?

Initial experience in the field of THV interventions is largely limited to the aortic valve and has shown that most procedures are well tolerated, have high procedural success and low in-hospital and early mortality. However, anatomical, mechanical and functional features of VHD in patient under CF-LVAD support introduce unique challenges which are still managed on a case-by-case basis due to the lack of evidence-based guidelines. These challenges are both clinical (early recognition, decision to intervene, optimal timing, balancing the risks and benefits, etc.) and technical in nature. In particular, the continuous flow, annular dilation, and absent annular calcifications encountered in CF-LVAD patients can precipitate device migration in TAVR. However, delays in management may lead to refractory heart failure. Data from case reports have suggested that individual patient-tailored considerations for device selection and choice of access are of paramount importance for the success of the procedure. This certainly requires a multidisciplinary heart team approach, involving specialists from every field of the cardiac sciences (imaging, heart failure and interventional cardiologists, intensivist, cardiac anesthesiologist, cardiac surgeon, LVAD coordinator, etc.). As more data will become available, guidelines will certainly evolve to address the management of VHD not only at the time of LVAD implantation but also after LVAD initiation. They will probably also incorporate guidance about transcatheter therapies as part of that management.

Optimal THV device selection and sizing algorithms are not well described at the present time and will be an important topic for further study. Procedural techniques, including the use of rapid pacing and CF-LVAD pump speed modulation to optimize THV stability during deployment will also require further refinement. Therefore, there is a crucial need to gather more data from multicenter registries or prospective trials.

Advances in THV technology will continue to address life threatening complications such as device migration which is a unique challenge in CF-LVAD patients due to the combined effects of hydrodynamic forces exerted by the LVAD, the absence of annular calcifications and the presence of ventricular or aortic root dilation. On the other hand, the accumulation of patient-specific data will be an opportunity to develop patient-specific simulation-based planning to help predict outcomes after THV interventions in this high-risk patient population. Promising computational models have already been applied to various branches of percutaneous cardiac procedures (127–129).

## 7. Conclusion

Patients under CF-LVAD who require intervention for VHD are considered poor reoperative candidates. The rise of multiple transcatheter technologies therefore represents an appealing therapeutic avenue for these patients. A growing body of evidence has shown that THV procedures are feasible and safe in patients with CF-LVAD when adequate perioperative imaging and a tailored interventional strategy are adopted. However, the lack of data on mid- to long-term outcomes and the occurrence of distinct complications such as device migration, valve thrombosis or hemolysis remain of concern with these approaches (Table 1). Future studies assessing larger patient cohorts are required to sufficiently evaluate the efficacy of these techniques.

## References

[1] Takeda K Takayama H Kalesan B Uriel N Colombo PC Jorde UP Long-term outcome of patients on continuous-flow left ventricular assist device support. J Thorac Cardiovasc Surg. (2014) 148(4):1606–14. 10.1016/j.jtcvs.2014.04.009 25260275

[2] Dharmavaram N Hess T Jaeger H Smith J Hermsen J Murray D National trends in heart donor usage rates: are we efficiently transplanting more hearts? J Am Heart Assoc. (2021) 10(15):e019655. 10.1161/JAHA.120.019655 34315285 PMC8475695

[3] Wang TS Hernandez AF Felker GM Milano CA Rogers JG Patel CB. Valvular heart disease in patients supported with left ventricular assist devices. Circ Heart Fail. (2014) 7(1):215–22. 10.1161/CIRCHEARTFAILURE.113.000473 24449812

[4] Naganuma M Akiyama M Sasaki K Maeda K Ito K Suzuki T Aortic insufficiency causes symptomatic heart failure during left ventricular assist device support. Tohoku J Exp Med. (2021) 255(3):229–37. 10.1620/tjem.255.229 34789593

[5] Tanaka A Kitahara H Onsager D Song T Raikhelkar J Kim G Impact of residual valve disease on survival after implantation of left ventricular assist devices. Ann Thorac Surg. (2018) 106(6):1789–96. 10.1016/j.athoracsur.2018.06.075 30148976

[6] Feldman D Pamboukian SV Teuteberg JJ Birks E Lietz K Moore SA The 2013 international society for heart and lung transplantation guidelines for mechanical circulatory support: executive summary. J Heart Lung Transplant. (2013) 32(2):157–87. 10.1016/j.healun.2012.09.013 23352391

[7] Riebandt J Schaefer A Wiedemann D Schloglhofer T Laufer G Sandner S Concomitant cardiac surgery procedures during left ventricular assist device implantation: single-centre experience. Ann Cardiothorac Surg. (2021) 10(2):248–54. 10.21037/acs-2020-cfmcs-30 33842219 PMC8033261

[8] Cribier A Eltchaninoff H Bash A Borenstein N Tron C Bauer F Percutaneous transcatheter implantation of an aortic valve prosthesis for calcific aortic stenosis: first human case description. Circulation. (2002) 106(24):3006–8. 10.1161/01.CIR.0000047200.36165.B8 12473543

[9] Banovic M Bartunek J Nikolic SD Vukcevic V Aleksandric S Iung B. Percutaneous treatment of aortic valve disease: contemporary overview and future trends. Curr Pharm Des. (2017) 23(31):4687–95. 10.2174/1381612822666161018162022 27774907

[10] Partida RA Elmariah S. Transcatheter mitral valve interventions: current therapies and future directions. Curr Treat Options Cardiovasc Med. (2017) 19(5):32. 10.1007/s11936-017-0538-2 28364394

[11] Asmarats L Puri R Latib A Navia JL Rodes-Cabau J. Transcatheter tricuspid valve interventions: landscape, challenges, and future directions. J Am Coll Cardiol. (2018) 71(25):2935–56. 10.1016/j.jacc.2018.04.031 29929618

[12] Driesen BW Warmerdam EG Sieswerda GJ Meijboom FJ Molenschot MMC Doevendans PA Percutaneous pulmonary valve implantation: current Status and future perspectives. Curr Cardiol Rev. (2019) 15(4):262–73. 10.2174/1573403X15666181224113855 30582483 PMC8142351

[13] D'Ancona G Pasic M Buz S Drews T Dreysse S Hetzer R TAVI For pure aortic valve insufficiency in a patient with a left ventricular assist device. Ann Thorac Surg. (2012) 93(4):e89–91. 10.1016/j.athoracsur.2011.11.019 22450111

[14] Pal JD Klodell CT John R Pagani FD Rogers JG Farrar DJ Low operative mortality with implantation of a continuous-flow left ventricular assist device and impact of concurrent cardiac procedures. Circulation. (2009) 120(11 Suppl):S215–9. 10.1161/CIRCULATIONAHA.108.844274 19752370

[15] Jorde UP Uriel N Nahumi N Bejar D Gonzalez-Costello J Thomas SS Prevalence, significance, and management of aortic insufficiency in continuous flow left ventricular assist device recipients. Circ Heart Fail. (2014) 7(2):310–9. 10.1161/CIRCHEARTFAILURE.113.000878 24415682

[16] Pak SW Uriel N Takayama H Cappleman S Song R Colombo PC Prevalence of de novo aortic insufficiency during long-term support with left ventricular assist devices. J Heart Lung Transplant. (2010) 29(10):1172–6. 10.1016/j.healun.2010.05.018 20619680

[17] Cowger J Pagani FD Haft JW Romano MA Aaronson KD Kolias TJ. The development of aortic insufficiency in left ventricular assist device-supported patients. Circ Heart Fail. (2010) 3(6):668–74. 10.1161/CIRCHEARTFAILURE.109.917765 20739615 PMC3089421

[18] Cowger J Rao V Massey T Sun B May-Newman K Jorde U Comprehensive review and suggested strategies for the detection and management of aortic insufficiency in patients with a continuous-flow left ventricular assist device. J Heart Lung Transplant. (2015) 34(2):149–57. 10.1016/j.healun.2014.09.045 25511746

[19] John R Mantz K Eckman P Rose A May-Newman K. Aortic valve pathophysiology during left ventricular assist device support. J Heart Lung Transplant. (2010) 29(12):1321–9. 10.1016/j.healun.2010.06.006 20674397

[20] Westaby S Bertoni GB Clelland C Nishinaka T Frazier OH. Circulatory support with attenuated pulse pressure alters human aortic wall morphology. J Thorac Cardiovasc Surg. (2007) 133(2):575–6. 10.1016/j.jtcvs.2006.10.014 17258608

[21] Bouabdallaoui N El-Hamamsy I Pham M Giraldeau G Parent MC Carrier M Aortic regurgitation in patients with a left ventricular assist device: a contemporary review. J Heart Lung Transplant. (2018) 37(11):1289–97. 10.1016/j.healun.2018.07.002 30197211

[22] Feldman CM Silver MA Sobieski MA Slaughter MS. Management of aortic insufficiency with continuous flow left ventricular assist devices: bioprosthetic valve replacement. J Heart Lung Transplant. (2006) 25(12):1410–2. 10.1016/j.healun.2006.10.004 17178333

[23] Cowger JA Aaronson KD Romano MA Haft J Pagani FD. Consequences of aortic insufficiency during long-term axial continuous-flow left ventricular assist device support. J Heart Lung Transplant. (2014) 33(12):1233–40. 10.1016/j.healun.2014.06.008 25108909

[24] Truby LK Garan AR Givens RC Wayda B Takeda K Yuzefpolskaya M Aortic insufficiency during contemporary left ventricular assist device support: analysis of the INTERMACS registry. JACC Heart Fail. (2018) 6(11):951–60. 10.1016/j.jchf.2018.07.012 30384913 PMC6217859

[25] Kormos RL Cowger J Pagani FD Teuteberg JJ Goldstein DJ Jacobs JP The society of thoracic surgeons intermacs database annual report: evolving indications, outcomes, and scientific partnerships. Ann Thorac Surg. (2019) 107(2):341–53. 10.1016/j.athoracsur.2018.11.011 30691584

[26] Morgan JA Brewer RJ Nemeh HW Murthy R Williams CT Lanfear DE Left ventricular reverse remodeling with a continuous flow left ventricular assist device measured by left ventricular end-diastolic dimensions and severity of mitral regurgitation. ASAIO J. (2012) 58(6):574–7. 10.1097/MAT.0b013e31826e4267 23103696

[27] Cruz Rodriguez JB Chatterjee A Pamboukian SV Tallaj JA Joly J Lenneman A Persistent mitral regurgitation after left ventricular assist device: a clinical conundrum. ESC Heart Fail. (2021) 8(2):1039–46. 10.1002/ehf2.12919 33471962 PMC8006607

[28] Milano CA Simeone AA Blue LJ Rogers JG. Presentation and management of left ventricular assist device inflow cannula malposition. J Heart Lung Transplant. (2011) 30(7):838–40. 10.1016/j.healun.2011.03.003 21489813

[29] Kassis H Cherukuri K Agarwal R Kanwar M Elapavaluru S Sokos GG Significance of residual mitral regurgitation after continuous flow left ventricular assist device implantation. JACC Heart Fail. (2017) 5(2):81–8. 10.1016/j.jchf.2016.09.014 28017353

[30] Ertugay S Kemal HS Kahraman U Engin C Nalbantgil S Yagdi T Impact of residual mitral regurgitation on right ventricular systolic function after left ventricular assist device implantation. Artif Organs. (2017) 41(7):622–7. 10.1111/aor.12831 27873344

[31] Jain R Truby LK Topkara VK. Residual mitral regurgitation in patients with left ventricular assist device support—an INTERMACS analysis. J Heart Lung Transplant. (2022) 41:1638–45. 10.1016/j.healun.2022.03.002 35379546

[32] Piacentino V 3rd Williams ML Depp T Garcia-Huerta K Blue L Lodge AJ Impact of tricuspid valve regurgitation in patients treated with implantable left ventricular assist devices. Ann Thorac Surg. (2011) 91(5):1342–6; discussion 6–7. 10.1016/j.athoracsur.2011.01.053 21457940

[33] Slaughter MS Pagani FD Rogers JG Miller LW Sun B Russell SD Clinical management of continuous-flow left ventricular assist devices in advanced heart failure. J Heart Lung Transplant. (2010) 29(4 Suppl):S1–39. 10.1016/j.healun.2010.01.011 20181499

[34] Noly PE Pagani FD Noiseux N Stulak JM Khalpey Z Carrier M Continuous-Flow left ventricular assist devices and valvular heart disease: a comprehensive review. Can J Cardiol. (2020) 36(2):244–60. 10.1016/j.cjca.2019.11.022 32036866

[35] Berthelot E Bauer F Eicher JC Flecher E Gellen B Guihaire J Pulmonary hypertension in chronic heart failure: definitions, advances, and unanswered issues. ESC Heart Fail. (2018) 5(5):755–63. 10.1002/ehf2.12316 30030912 PMC6165943

[36] Al-Kindi SG Farhoud M Zacharias M Ginwalla MB ElAmm CA Benatti RD Left ventricular assist devices or inotropes for decreasing pulmonary vascular resistance in patients with pulmonary hypertension listed for heart transplantation. J Card Fail. (2017) 23(3):209–15. 10.1016/j.cardfail.2016.06.421 27374840

[37] Nakanishi K Homma S Han J Takayama H Colombo PC Yuzefpolskaya M Prevalence, predictors, and prognostic value of residual tricuspid regurgitation in patients with left ventricular assist device. J Am Heart Assoc. (2018) 7(13). 10.1161/JAHA.118.008813 29937432 PMC6064878

[38] Westaby S. Tricuspid regurgitation in left ventricular assist device patients. Eur J Cardiothorac Surg. (2012) 41(1):217–8. 10.1016/j.ejcts.2011.06.019 21820908 PMC3241100

[39] Zhigalov K Szczechowicz M Mashhour A Kadyraliev BK Mkalaluh S Easo J Left ventricular assist device implantation with concomitant tricuspid valve repair: is there really a benefit? J Thorac Dis. (2019) 11(Suppl 6):S902–S12. 10.21037/jtd.2018.11.116 31183169 PMC6535483

[40] Dunlay SM Deo SV Park SJ. Impact of tricuspid valve surgery at the time of left ventricular assist device insertion on postoperative outcomes. ASAIO J. (2015) 61(1):15–20. 10.1097/MAT.0000000000000145 25238498 PMC4280315

[41] Nakazato T Yoshioka D Toda K Miyagawa S Kainuma S Kawamura T Impact of tricuspid regurgitation on late right ventricular failure in left ventricular assist device patients ∼can prophylactic tricuspid annuloplasty prevent late right ventricular failure? ∼. J Cardiothorac Surg. (2021) 16(1):99. 10.1186/s13019-021-01492-0 33879203 PMC8056678

[42] Song HK Gelow JM Mudd J Chien C Tibayan FA Hollifield K Limited utility of tricuspid valve repair at the time of left ventricular assist device implantation. Ann Thorac Surg. (2016) 101(6):2168–74. 10.1016/j.athoracsur.2016.03.040 27139368 PMC4877201

[43] Khan S Koerner MM Pae W Stephenson ER Weber H Brehm C Successful percutaneous transcatheter aortic valve replacement in multi-organ failure due to aortic bioprosthesis regurgitation in a patient with continuous-flow LVAD. J Heart Lung Transplant. (2013) 32(6):659–63. 10.1016/j.healun.2013.03.007 23701856

[44] Ali J Catarino P Abu-Omar Y. Transcatheter aortic valve implantation in patients with a left ventricular assist device: a word of caution. Eur J Cardiothorac Surg. (2020) 58(6):1309–10. 10.1093/ejcts/ezaa237 32766699

[45] Ong BH Chiam PT Sim DK Tan TE. Post-implantation transcatheter aortic valve migration in a left ventricular assist device patient with severe aortic insufficiency. Eur Heart J. (2014) 35(24):1616. 10.1093/eurheartj/eht516 24334716

[46] Phan K Haswell JM Xu J Assem Y Mick SL Kapadia SR Percutaneous transcatheter interventions for aortic insufficiency in continuous-flow left ventricular assist device patients: a systematic review and meta-analysis. ASAIO J. (2017) 63(2):117–22. 10.1097/MAT.0000000000000447 27676407

[47] Pal JD McCabe JM Dardas T Aldea GS Mokadam NA. Transcatheter aortic valve repair for management of aortic insufficiency in patients supported with left ventricular assist devices. J Card Surg. (2016) 31(10):654–7. 10.1111/jocs.12814 27487763

[48] Chung MJ Ganapathi AM Vora AN Schroder JN Kiefer TL Hughes GC. Valve-in-Ring transcatheter aortic valve replacement after left ventricular assist device therapy. Ann Thorac Surg. (2020) 109(3):e163–e5. 10.1016/j.athoracsur.2019.06.094 31445912

[49] Derryberry SL Williams RD Fredi JL Ball SK. Complete fusion of a percutaneous aortic valve placed after ventricular assist device. Interact Cardiovasc Thorac Surg. (2017) 25(2):329–30. 10.1093/icvts/ivx085 28475802

[50] Parry D Rao V Butany J Catrip J Wilson W McDonald M Transcatheter aortic valve implantation and left ventricular assist device: a word of caution. Ann Thorac Surg. (2014) 97(2):e41–2. 10.1016/j.athoracsur.2013.09.071 24484841

[51] Rao SD Jagasia D Anwaruddin S Birati EY. Transcatheter aortic valve replacement thrombosis in patient supported with durable left ventricular assist device. Catheter Cardiovasc Interv. (2020) 96(2):500–3. 10.1002/ccd.28742 31977150

[52] Meduri C Kauten J Vannan M Rajagopal V. First report of a simultaneous transcatheter mitral valve-in-valve and aortic valve replacement in a left ventricular assist device patient. Catheter Cardiovasc Interv. (2017) 90(3):526–9. 10.1002/ccd.26983 28295972

[53] Wilson W Goldraich L Parry D Cusimano R Rao V Horlick E. Cardiac arrest secondary to sudden LVAD failure in the setting of aortic valve fusion successfully managed with emergent transcatheter aortic valve replacement. Int J Cardiol. (2014) 171(2):e40–1. 10.1016/j.ijcard.2013.11.117 24387895

[54] Ribichini F Faggian G Pesarini G Milano A Gottin L Vassanelli C. Bail-out transcatheter aortic valve implantation to reduce severe acute aortic regurgitation in a failing homograft secondary to HeartMate II ventricular assistance device. Cardiovasc Revasc Med. (2014) 15(5):295–7. 10.1016/j.carrev.2013.12.002 24445212

[55] Kozarek K Minhaj MM Chaney MA D'Ancona G Pasic M Carrel T Transcatheter aortic valve replacement for left ventricular assist device-induced aortic insufficiency. J Cardiothorac Vasc Anesth. (2018) 32(4):1982–90. 10.1053/j.jvca.2018.01.013 29699845

[56] Danley MR Lensing C Eagle SS Kingeter MA. Transcatheter aortic valve replacement in patients with left ventricular assist devices and aortic regurgitation-single institution retrospective analysis. J Cardiothorac Vasc Anesth. (2022) 36(8 Pt A):2839–40. 10.1053/j.jvca.2022.03.020 35466018

[57] Bayram Z Dogan C Gurcu E Kaymaz C Ozdemir N. Development of acute severe right heart failure after transcatheter aortic valve implantation in patient with left ventricle assist device-acquired aortic regurgitation. Turk Kardiyol Dern Ars. (2020) 48(5):545–51. 10.5543/tkda.2020.39132 32633261

[58] Chang HH Chen PL Chen YH Leu HB Kuo CC Wu NY. Intraoperative ECMO assistance during TAVI for aortic insufficiency in an Asian patient with LVAD support. ESC Heart Fail. (2021) 8(4):3418–21. 10.1002/ehf2.13426 34085412 PMC8318486

[59] Rene AG Desai N Wald J Rame JE Frogel JK Anwaruddin S. Transfemoral transcatheter aortic valve replacement with a self-expanding valve for severe aortic regurgitation in a patient with left ventricular assist device. J Card Surg. (2017) 32(11):741–5. 10.1111/jocs.13238 29178215

[60] Yehya A Rajagopal V Meduri C Kauten J Brown M Dean L Short-term results with transcatheter aortic valve replacement for treatment of left ventricular assist device patients with symptomatic aortic insufficiency. J Heart Lung Transplant. (2019) 38(9):920–6. 10.1016/j.healun.2019.03.001 30898555

[61] Dhillon AS Jones BM Hodson RW Korngold EC. Transcatheter aortic valve replacement for severe aortic regurgitation in patients with a left ventricular assist device. J Invasive Cardiol. (2022) 34(5):E369–E73. 35343915 10.25270/jic/21.00212

[62] van der Werf HW Schurer RA Vonck TE Poelman JE Klungel AA Cernak V Emergency transcatheter aortic valve implantation in patients with severe aortic regurgitation and a left ventricle assist device: a case report and systematic review. Eur Heart J Acute Cardiovasc Care. (2017) 6(8):719–27. 10.1177/2048872616652310 27245700

[63] Al Emam AR Barton D Um J Pavlides G. Valve in valve trans-catheter aortic valve replacement followed by LVAD deactivation in the setting of recovered systolic function. Curr Cardiol Rev. (2020) 16(1):77–80. 10.2174/1573403X15666190509082833 31072295 PMC7393596

[64] Gomes B Bekeredjian R Leuschner F Ehlermann P Schmack B Ruhparwar A Transfemoral aortic valve replacement for severe aortic valve regurgitation in a patient with a pulsatile-flow biventricular assist device. ESC Heart Fail. (2019) 6(1):217–21. 10.1002/ehf2.12384 30479049 PMC6351890

[65] Yap J Stripe BR Wong GB Smith TW Southard JA. Technical considerations for TAVR in the treatment of stentless bioprosthetic aortic valve insufficiency in LVAD patients. J Invasive Cardiol. (2020) 32(6):E174. 32479422 10.25270/jic/19.00390

[66] Vavalle JP Kiefer TL Milano CA Harrison JK Hughes GC. Transcatheter aortic valve replacement performed via left ventricular assist device inflow cannula. Circ Heart Fail. (2014) 7(3):544–6. 10.1161/CIRCHEARTFAILURE.114.001232 24847132

[67] Bjelic M Ayers B Ling FS Prasad SM Gosev I. Transcatheter aortic valve replacement in left ventricular assist device patient-overcoming the complications with transapical approach and circulatory arrest. J Card Surg. (2021) 36(1):403–5. 10.1111/jocs.15199 33225501

[68] Mondal S Dawood M Bandyopadhyay D Taylor BS Tanaka K Gupta A. Transcatheter aortic valve replacement: a potential option for aortic insufficiency management in patients with left ventricular assist device. Int J Cardiol Heart Vasc. (2020) 26:100425. 10.1016/j.ijcha.2019.100425 31763439 PMC6864334

[69] Kar B Prathipati P Jumean M Nathan SS Gregoric ID. Management of aortic insufficiency using transcatheter aortic valve replacement in patients with left ventricular assist device support. ASAIO J. (2020) 66(6):e82–e6. 10.1097/MAT.0000000000001053 31425270

[70] Rudisill KG Smallfield MC Shah KB Quader MA Bhardwaj HL Gertz ZM. Transcatheter heart valve thrombosis in a patient with a left ventricular assist device. Circ Heart Fail. (2020) 13(9):e007112. 10.1161/CIRCHEARTFAILURE.120.007112 32842759

[71] FE MH Wellmann P von Dossow V Massberg S Hagl C Schramm R Rescue TAVI for aortic regurgitation after left ventricular assist device implantation following preoperative impella(R) support. J Heart Valve Dis. (2017) 26(5):603–5. 29762934

[72] Beukian S Lala A Dangas G Tang GHL Anyanwu A Moss N Subacute aortic root and valve thrombosis following transcatheter aortic valve replacement in a left ventricular assist device patient: from one problem to the next. CASE (Phila). (2021) 5(2):97–100. 10.1016/j.case.2020.12.005 33912777 PMC8071813

[73] Sarwari H Schaefer A Barten MJ Conradi L. TAVI Using a self-expandable device for aortic regurgitation following LVAD implantation. Thorac Cardiovasc Surg Rep. (2019) 8(1):e33–e6. 10.1055/s-0039-1698412 31692909 PMC6828560

[74] Ranard LS Kaple R Khalique OK Agarwal V Bellumkonda L Bonde P First transfemoral implantation of a novel transcatheter valve in an LVAD patient with aortic insufficiency. JACC Case Rep. (2021) 3(17):1806–10. 10.1016/j.jaccas.2021.08.026 34917959 PMC8642726

[75] Zaidi SH Minhas AMK Sagheer S ManeshGangwani K Dani SS Goel SS Clinical outcomes of transcatheter aortic valve replacement (TAVR) vs. Surgical aortic valve replacement (SAVR) in patients with durable left ventricular assist device (LVAD). Curr Probl Cardiol. (2022) 47(10):101313. 10.1016/j.cpcardiol.2022.101313 35817155

[76] Bietry R Balsam LB Saric M McElhinney DB Katz S Deanda A Jr. Percutaneous intervention for recurrent aortic insufficiency in a patient with a left ventricular assist device and a centrally oversewn aortic valve. Circ Heart Fail. (2013) 6(4):e43–4. 10.1161/CIRCHEARTFAILURE.113.000301 23861507

[77] Parikh KS Mehrotra AK Russo MJ Lang RM Anderson A Jeevanandam V Percutaneous transcatheter aortic valve closure successfully treats left ventricular assist device-associated aortic insufficiency and improves cardiac hemodynamics. JACC Cardiovasc Interv. (2013) 6(1):84–9. 10.1016/j.jcin.2012.08.021 23347865

[78] Freed BH Paul JD Bhave NM Russo MJ Jeevanandam V Lang RM Percutaneous transcatheter closure of the native aortic valve to treat de novo aortic insufficiency after implantation of a left ventricular assist device. JACC Cardiovasc Interv. (2012) 5(3):358–9. 10.1016/j.jcin.2011.11.012 22440504

[79] Russo MJ Freed BH Jeevanandam V Hashmi M Paul JD Anderson A Percutaneous transcatheter closure of the aortic valve to treat cardiogenic shock in a left ventricular assist device patient with severe aortic insufficiency. Ann Thorac Surg. (2012) 94(3):985–8. 10.1016/j.athoracsur.2012.01.089 22916753

[80] Feider A Dhawan R Chaney M. Percutaneous closure of an incompetent aortic valve using an occluder device in a patient with left ventricular assist device. Anesth Analg. (2013) 117(5):1078–80. 10.1213/ANE.0b013e3182a4e433 24029857

[81] Sauer AJ Davidson CJ McGee EC Jr. Percutaneous closure of the aortic valve as a bridge to heart transplantation to treat severe aortic insufficiency after ventricular assist device. Catheter Cardiovasc Interv. (2015) 86(2):E103–6. 10.1002/ccd.25584 24975484

[82] Grohmann J Blanke P Benk C Schlensak C. Trans-catheter closure of the native aortic valve with an amplatzer occluder to treat progressive aortic regurgitation after implantation of a left-ventricular assist device. Eur J Cardiothorac Surg. (2011) 39(6):e181–3. 10.1016/j.ejcts.2011.01.036 21377889

[83] Rajagopal K Daneshmand MA Patel CB Ganapathi AM Schechter MA Rogers JG Natural history and clinical effect of aortic valve regurgitation after left ventricular assist device implantation. J Thorac Cardiovasc Surg. (2013) 145(5):1373–9. 10.1016/j.jtcvs.2012.11.066 23312101

[84] Alkhouli M Melito B Ling FS. Percutaneous aortic valve closure for patients with left ventricular assist device-associated aortic insufficiency. Catheter Cardiovasc Interv. (2016) 88(7):1170–3. 10.1002/ccd.26314 26514340

[85] Guvenc TS Ertas G Sargin M. Percutaneous closure of aortic valve complicated by right ventricular failure in a patient with left ventricular assist device. Artif Organs. (2016) 40(4):416–8. 10.1111/aor.12561 27076204

[86] Zacharias M Dhingra R. Percutaneous treatment of severe aortic insufficiency in a patient with left ventricular assist device: friend or foe. JACC Cardiovasc Interv. (2015) 8(5):750–1. 10.1016/j.jcin.2014.10.030 25946449

[87] Nicolo F Montalto A Musto C Comisso M Lio A Musumeci F. Percutaneous aortic valve closure in patient with left ventricular assist device and dilated aortic Annulus. Ann Thorac Surg. (2020) 109(1):e25–e7. 10.1016/j.athoracsur.2019.04.081 31207247

[88] Retzer EM Sayer GT Fedson SE Nathan S Jeevanandam V Friant J Predictors of survival following trans-catheter aortic valve closure for left ventricular assist device associated aortic insufficiency. Catheter Cardiovasc Interv. (2016) 87(5):971–9. 10.1002/ccd.26280 26527571

[89] Pollak P Lim DS Kern J. Management of severe aortic regurgitation in a patient with cardiogenic shock using a percutaneous left ventricular assist device and transcatheter occlusion of the failed aortic valve homograft as a bridge to surgical valve replacement. Catheter Cardiovasc Interv. (2014) 83(1):E141–5. 10.1002/ccd.24993 23674479

[90] Cork DP Adamson R Gollapudi R Dembitsky W Jaski B. Percutaneous repair of postoperative mitral regurgitation after left ventricular assist device implant. Ann Thorac Surg. (2018) 105(2):e45–e6. 10.1016/j.athoracsur.2017.09.042 29362189

[91] Raghunathan D Arora A Aman W Nathan S Jumean M Gregoric ID Transcatheter mitral valve repair for severe,symptomatic mitral regurgitation in patients with left ventricular assist devices. Methodist Debakey Cardiovasc J. (2021) 17(1):e1–4. 10.14797/DPYF8504 34104329 PMC8158451

[92] Tanveer Ud Din M Minhas AMK Muslim MO Wazir MHK Dani SS Goel SS Outcomes of MitraClip and surgical mitral valve repair in patients with left ventricular assist device. Am J Cardiol. (2022) 173:143–5. 10.1016/j.amjcard.2022.03.012 35437161

[93] Andreas M Russo M Werner P Schneider M Wittmann F Scherzer S Transcatheter edge-to-edge tricuspid repair for recurrence of valvular regurgitation after left ventricular assist device and tricuspid ring implantation. ESC Heart Fail. (2020) 7(3):915–9. 10.1002/ehf2.12577 32144947 PMC7261524

[94] Edelman JJ Khan JM Rogers T Shults C Satler LF Ben D II Valve-in-Valve TAVR: state-of-the-art review. Innovations (Phila). (2019) 14(4):299–310. 10.1177/1556984519858020 31328655

[95] Franzone A Piccolo R Siontis GCM Lanz J Stortecky S Praz F Transcatheter aortic valve replacement for the treatment of pure native aortic valve regurgitation: a systematic review. JACC Cardiovasc Interv. (2016) 9(22):2308–17. 10.1016/j.jcin.2016.08.049 28026742

[96] Fine NM Park SJ Stulak JM Topilsky Y Daly RC Joyce LD Proximal thoracic aorta dimensions after continuous-flow left ventricular assist device implantation: longitudinal changes and relation to aortic valve insufficiency. J Heart Lung Transplant. (2016) 35(4):423–32. 10.1016/j.healun.2015.10.029 26632029

[97] Jiang J Liu X He Y Xu Q Zhu Q Jaiswal S Transcatheter aortic valve replacement for pure native aortic valve regurgitation: a systematic review. Cardiology. (2018) 141(3):132–40. 10.1159/000491919 30517917

[98] Haddad A Arwani R Altayar O Sawas T Murad MH de Marchena E. Transcatheter aortic valve replacement in patients with pure native aortic valve regurgitation: a systematic review and meta-analysis. Clin Cardiol. (2019) 42(1):159–66. 10.1002/clc.23103 30350358 PMC6436518

[99] Sinning JM Werner N Nickenig G Grube E. Next-generation transcatheter heart valves: current trials in Europe and the USA. Methodist Debakey Cardiovasc J. (2012) 8(2):9–12. 10.14797/mdcj-8-2-9 22891121 PMC3405796

[100] Baum C Seiffert M Treede H Reichenspurner H Deuse T. Concomitant transcatheter aortic valve and left ventricular assist device implantation. ASAIO J. (2013) 59(1):90–2. 10.1097/MAT.0b013e318277a835 23271392

[101] Seiffert M Bader R Kappert U Rastan A Krapf S Bleiziffer S Initial German experience with transapical implantation of a second-generation transcatheter heart valve for the treatment of aortic regurgitation. JACC Cardiovasc Interv. (2014) 7(10):1168–74. 10.1016/j.jcin.2014.05.014 25129672

[102] Schlingloff F Schafer U Frerker C Schmoeckel M Bader R. Transcatheter aortic valve implantation of a second-generation valve for pure aortic regurgitation: procedural outcome, haemodynamic data and follow-up. Interact Cardiovasc Thorac Surg. (2014) 19(3):388–93. 10.1093/icvts/ivu155 24893871

[103] Ten Berg J Sibbing D Rocca B Van Belle E Chevalier B Collet JP Management of antithrombotic therapy in patients undergoing transcatheter aortic valve implantation: a consensus document of the ESC working group on thrombosis and the European association of percutaneous cardiovascular interventions (EAPCI), in collaboration with the ESC council on valvular heart disease. Eur Heart J. (2021) 42(23):2265–9. 10.1093/eurheartj/ehab196 33822924

[104] Otto CM Nishimura RA Bonow RO Carabello BA Erwin JP 3rd Gentile F 2020 ACC/AHA guideline for the management of patients with valvular heart disease: executive summary: a report of the American college of cardiology/American heart association joint committee on clinical practice guidelines. Circulation. (2021) 143(5):e35–71. 10.1161/CIR.0000000000000932 33332149

[105] Dogan G Hanke JS Ricklefs M Chatterjee A Feldmann C Mashaqi B Mitraclip procedure prior to left ventricular assist device implantation. J Thorac Dis. (2018) 10(Suppl 15):S1763–S8. 10.21037/jtd.2018.05.36 30034850 PMC6035960

[106] Ammirati E Van De Heyning CM Musca F Brambatti M Perna E Cipriani M Safety of centrifugal left ventricular assist device in patients previously treated with MitraClip system. Int J Cardiol. (2019) 283:131–3. 10.1016/j.ijcard.2019.02.039 30833105

[107] Kreusser MM Hamed S Weber A Schmack B Volz MJ Geis NA Mitraclip implantation followed by insertion of a left ventricular assist device in patients with advanced heart failure. ESC Heart Fail. (2020) 7:3891–900. 10.1002/ehf2.12982 PMC7754960 33107214

[108] Fox H Gyoten T Rojas SV Deutsch MA Schramm R Rudolph V Safety, mortality, and hemodynamic impact of patients with MitraClip undergoing left ventricular assist device implantation. J Cardiovasc Transl Res. (2022) 15(3):676–86. 10.1007/s12265-021-10178-w 34713397 PMC9213377

[109] Godino C Munafo A Scotti A Estevez-Loureiro R Portoles Hernandez A Arzamendi D Mitraclip in secondary mitral regurgitation as a bridge to heart transplantation: 1-year outcomes from the international MitraBridge registry. J Heart Lung Transplant. (2020) 39(12):1353–62. 10.1016/j.healun.2020.09.005 33008726

[110] Tanaka S Imamura T Ueno H Kinugawa K. Mitraclip or ventricular assist device? Int Heart J. (2020) 61(6):1303–6. 10.1536/ihj.20-333 33191352

[111] Godino C Munafo AR Scotti A Saia F. Strategies for bridge to heart transplantation: percutaneous mitral valve repair and LVAD. J Heart Lung Transplant. (2021) 40(6):536–7. 10.1016/j.healun.2021.03.012 33849771

[112] Bertrand PB Namasivayam M. Left ventricular assist device after percutaneous mitral valve repair: can we go there? Int J Cardiol. (2019) 288:55–6. 10.1016/j.ijcard.2019.03.052 30954287

[113] Pappalardo F Morici N Colombo PC. Mitrabridge and left ventricular assist device: crossing dangerous bridges. J Heart Lung Transplant. (2021) 40(4):316–7. 10.1016/j.healun.2021.01.004 33518453

[114] Kadado AJ Islam A. Iatrogenic atrial septal defect following the MitraClip procedure: a state-of-the-art review. Catheter Cardiovasc Interv. (2021) 97(7):E1043–E52. 10.1002/ccd.29149 32710470

[115] Shah NR Cevik C Hernandez A Gregoric ID Frazier OH Stainback RF. Transthoracic echocardiography of the HeartWare left ventricular assist device. J Card Fail. (2012) 18(9):745–8. 10.1016/j.cardfail.2012.06.529 22939044

[116] Ben Ali W Ruf T Perrin N Bouhout I Fam N Kresoja KP Indications, limitations, and development of tricuspid valve interventions in adults. Can J Cardiol. (2021) 38:S66–S78. 10.1016/j.cjca.2021.08.013 34464691

[117] Kolte D Elmariah S. Transcatheter tricuspid valve therapy. Curr Treat Options Cardiovasc Med. (2019) 21(6):26. 10.1007/s11936-019-0730-7 31104153

[118] Taramasso M Alessandrini H Latib A Asami M Attinger-Toller A Biasco L Outcomes after current transcatheter tricuspid valve intervention: mid-term results from the international TriValve registry. JACC Cardiovasc Interv. (2019) 12(2):155–65. 10.1016/j.jcin.2018.10.022 30594510

[119] Fucci C Lorusso R Totaro P Ceconi C Nardi M Coletti G The triple-orifice repair: a new technique for the treatment of mitral regurgitation in severe Barlow's Disease. Ital Heart J. (2004) 5(3):238–40. 15119509

[120] Alfieri O De Bonis M Lapenna E Agricola E Quarti A Maisano F. The “clover technique” as a novel approach for correction of post-traumatic tricuspid regurgitation. J Thorac Cardiovasc Surg. (2003) 126(1):75–9. 10.1016/S0022-5223(03)00204-6 12878941

[121] Lurz P Stephan von Bardeleben R Weber M Sitges M Sorajja P Hausleiter J Transcatheter edge-to-edge repair for treatment of tricuspid regurgitation. J Am Coll Cardiol. (2021) 77(3):229–39. 10.1016/j.jacc.2020.11.038 33478646

[122] Goldberg YH Ho E Chau M Latib A. Update on transcatheter tricuspid valve replacement therapies. Front Cardiovasc Med. (2021) 8:619558. 10.3389/fcvm.2021.619558 33659278 PMC7917079

[123] Zaghloul A Iorgoveanu C Desai A Silkowski M Balakumaran K. Prosthetic tricuspid valve thrombosis. Cureus. (2018) 10(7):e2928. 10.7759/cureus.2928 30197850 PMC6126703

[124] Rali AS Taduru SS Tran LE Ranka S Schlendorf KH Barker CM Transcatheter aortic valve replacement and surgical aortic valve replacement outcomes in left ventricular assist device patients with aortic insufficiency. Card Fail Rev. (2022) 8:e30. 10.15420/cfr.2022.21 36644645 PMC9819997

[125] Gondi KT Tam MC Chetcuti SJ Pagani FD Grossman PM Deeb GM Transcatheter aortic valve replacement for left ventricular assist device–related aortic regurgitation: the Michigan medicine experience. J Soci Cardiovasc Angiography & Interven. (2023) 2(1). 10.1016/j.jscai.2022.100530 PMC11307432 39132542

[126] Gyoten T Morshuis M Fox H Deutsch MA Hakim-Meibodi K Schramm R Secondary aortic valve replacement in continuous flow left ventricular assist device therapy. Artif Organs. (2021) 45(7):736–41. 10.1111/aor.13906 33432621

[127] de Jaegere P Rocatello G Prendergast BD de Backer O Van Mieghem NM Rajani R. Patient-specific computer simulation for transcatheter cardiac interventions: what a clinician needs to know. Heart. (2019) 105(Suppl 2):s21–s7. 10.1136/heartjnl-2018-313514 30846521

[128] Perrin N Mondesert B Thibodeau-Jarry N Pierre-Mongeon F Rousseau-Saine N Ibrahim R Simulation-based planning of transcatheter left atrial appendage occlusion. EuroIntervention. (2022) 18(3):233–4. 10.4244/EIJ-D-21-00731 35080197 PMC9912970

[129] El Faquir N De Backer O Bosmans J Rudolph T Buzzatti N Bieliauskas G Patient-Specific computer simulation in TAVR with the self-expanding evolut R valve. JACC Cardiovasc Interv. (2020) 13(15):1803–12. 10.1016/j.jcin.2020.04.018 32682679

